# Development and refinement of the Clinical Global Impression of Improvement for Non-seizure Symptoms measure in Dravet syndrome and Lennox-Gastaut syndrome

**DOI:** 10.1186/s41687-024-00829-2

**Published:** 2025-02-21

**Authors:** J. Scott Andrews, Drishti Shah, Alise Nacson, Tara Symonds, Sophie Hughes, Mahnaz Asgharnejad, Arturo Benitez, Lara Sams

**Affiliations:** 1 Takeda Development Center Americas, Inc., Cambridge, MA USA; 2 Clinical Outcomes Solutions, Chicago, IL USA; 3 Clinical Outcomes Solutions, Folkestone, UK

**Keywords:** Epilepsy, Dravet syndrome, Lennox-Gastaut syndrome, CGI-I Non-seizure Symptoms

## Abstract

**Background:**

Dravet syndrome (DS) and Lennox-Gastaut syndrome (LGS) are rare, severe, childhood-onset developmental and epileptic encephalopathies characterized by treatment-resistant epilepsy and varying intellectual disability levels. Clinical outcome assessments (COAs) describe how patients feel, function, or survive, thus providing valuable information on a therapy’s efficacy and impact. Individuals with DS or LGS are heterogeneous, and many have limited verbal abilities and intellectual disability. Existing epilepsy-specific COA measures are unsuitable for DS and LGS clinical trials as many items demonstrate floor effects in these populations. As patients often cannot self-report symptoms, caregiver feedback on the measures’ relevance and understandability is critical when developing COAs to ensure their suitability for the intended population, and that caregivers can help clinicians complete the measures when necessary.

**Methodology:**

We aimed to develop a novel clinician-reported outcomes measure, to be completed in consultation with caregivers at clinic visits, to assess non-seizure symptoms in individuals with DS or LGS using a Clinical Global Impression of Improvement (CGI–I) approach: the CGI-I Non-seizure Symptoms measure. A 13-item initial draft measure was reviewed by experts in a three-round Delphi panel to confirm each item’s relevance and refine descriptions, reduce overlap, and limit respondent burden.

**Results:**

Following panel review, three items reached consensus (≥70% agreement of no revision required) and were included in the final measure: communication, alertness, and disruptive behaviors. To ensure caregivers can help clinicians complete the measure, and to establish levels of change in each item domain considered meaningful from their perspective, the three-item measure was cognitively debriefed with caregivers of individuals with DS or LGS. Caregivers showed that each item was understandable by describing their child using the descriptions provided in the measure and reported that items were relevant or important to assess in DS or LGS. Most caregivers reported that even a minimal change to their child’s condition in each domain would be meaningful to them and their child.

**Conclusions:**

This CGI-I Non-seizure Symptoms measure represents relevant non-seizure outcomes considered important to individuals with DS or LGS and their families. The systematic development and refinement approach presented here supports its use in DS and LGS clinical trials.

**Supplementary information:**

The online version contains supplementary material available at 10.1186/s41687-024-00829-2.

## Background

Dravet syndrome (DS) and Lennox-Gastaut syndrome (LGS) are childhood-onset, rare developmental and epileptic encephalopathies (DEEs), characterized by treatment-resistant seizures, varying levels of developmental delay and/or intellectual disability, and a range of social, behavioral, and communication problems [1, 2]. Individuals with DS or LGS often require a lifetime of receiving care owing to the highly disabling symptom combination of epilepsy and intellectual and developmental disability (IDD), contributing to a substantial burden and impact on caregivers and families [3, 4].

For individuals with DS, onset is typically in the first year of life [5]. Incidence is ~2.2–6.5 per 100,000 and prevalence is ~1.2–6.5 per 100,000 people [5, 6]. Language impairment, poor fine motor abilities, lack of attention, and restlessness are common traits [7]. Frequent seizures, behavioral deterioration, and appearance of neurological signs typically occur between ages of 1 and 5 years [7]. Seizure types may change during childhood, with mental development/behavior improving after age 5 years [7]. LGS onset is typically between ages 18 months and 8 years [8]. Incidence is ~1.9 in 100,000 and prevalence is ~2.9–28 per 100,000 people [4]. Cognitive impairment is often present at diagnosis with serious intellectual disabilities observed within 5 years [2]. During childhood/adolescence, hyperactivity, aggression, autistic behaviors, and sleep disorders are common traits [8]. Typically, LGS persists into adulthood with individuals experiencing frequent atypical absence and tonic seizures [8].

Clinical outcome assessment (COA) measures are used to determine how a patient feels, functions, or survives [9]. In clinical trials, COAs can define efficacy endpoints and provide information on how well therapies perform [10]. Although there are many existing general epilepsy-specific COA measures, they are not appropriate for use in individuals with DS or LGS owing to the heterogeneity of these populations with respect to level of intellectual disability, verbal communication abilities, and functional status. This heterogeneity can make evaluation of non-seizure symptoms in affected individuals challenging, especially as they are often unable to reliably self-report, and improvements that could be considered clinically meaningful in these populations may involve an incrementally small change, which existing measures fail to capture [11]. Additionally, many COA measures used in studies of DS, LGS, and other similar epileptic conditions contain items that are not relevant to children with disabilities [12]. With these factors in mind, there is an unmet need for a COA measure that captures the complexities of DS and LGS and addresses the limitations of existing COA measures.

The use of a Clinical Global Impression (CGI) of Improvement (CGI-I) scale is a widely accepted approach for capturing an individual’s change in status after initiating experimental treatment in clinical trials [13]. Changes are assessed using the standard 7-point Likert scale for global impression items: 1-very much improved; 2-much improved; 3-minimally improved; 4-no change; 5-minimally worse; 6-much worse; and 7-very much worse [14].

CGI scales are commonly used in combination with disease-specific measures; however, disease-specific measures are not always available, particularly for rare conditions. In these instances, CGI scales have been adapted to include items evaluating global improvement in domains relevant to a particular condition in addition to “overall” [15]. For example, to account for change in a complex, heterogeneous population, a CGI-I-based approach has previously been adapted for use in schizoaffective disorder [16, 17], bipolar disorder [18], Rett syndrome [19], Prader-Willi syndrome [20], Angelman syndrome [15], and in DEEs [21].

Here, we describe the development and refinement of the CGI-I Non-seizure Symptoms measure, a disorder-specific measure based on the CGI-I for assessment of non-seizure symptoms in individuals with DS or LGS. This clinician-reported outcome (ClinRO) measure was developed specifically for use in clinical trials to allow a within-patient approach to assessing changes observed over time. Clinical notes and a description of the patient’s status are recorded at baseline and added to the electronic case report form for consultation at future assessments (as an alternative to recording a baseline severity score). Establishing a baseline description of the study participant before treatment initiation enables clinicians/caregivers to make an informed assessment of change at subsequent study visits and helps mitigate potential recall bias effects. The CGI-based approach was chosen because it is intuitive to use and interpret, may be sensitive to change in a population with heterogeneous disease presentation, and is flexible enough to detect improvement and worsening in individuals with profound intellectual disabilities.

## Material and methods

**Fig. 1:**
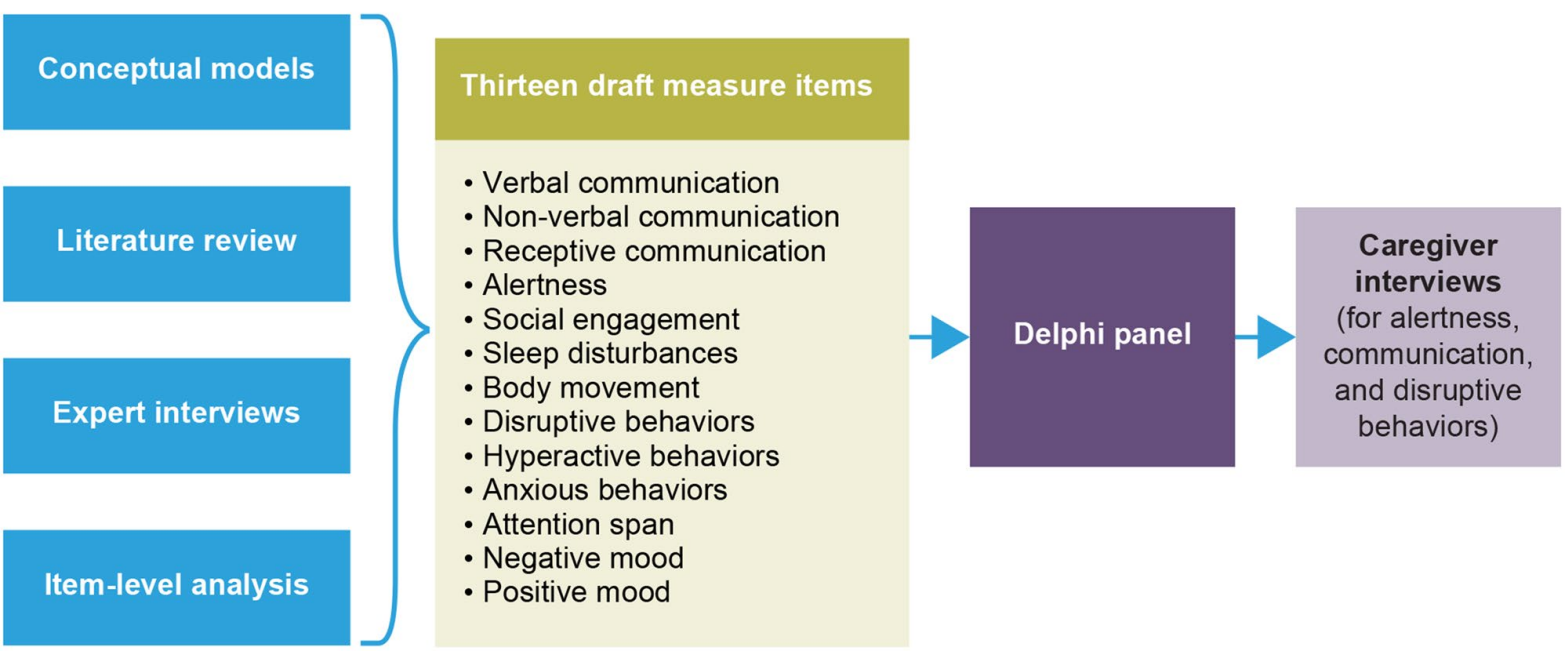
Study workflow: developing a draft CGI-I Non-seizure Symptoms measure for individuals with DS and LGS. *CGI-I* Clinical Global Impression of Improvement, *DS* Dravet syndrome, *LGS* Lennox-Gastaut syndrome

An overview of the iterative stages in the study process is provided in Fig. 1. Preliminary stages providing the basis of the draft CGI-I Non-seizure Symptoms measure comprised development of conceptual models, a targeted literature review of existing COA measures, expert interviews, and an item-level analysis of data from the phase 2 ELEKTRA clinical trial. Following these initial stages, a Delphi panel was convened to refine the draft CGI-I Non-seizure Symptoms measure and ensure it was acceptable to clinicians. The final CGI-I Non-seizure Symptoms measure was then debriefed with caregivers of individuals with DS or LGS.

### Preliminary analysis to aid draft measure development

Patient-centered conceptual models were developed based on the literature as well as interviews with expert clinicians and patient advocates (PAs), and include the key signs and symptoms of DS and LGS and the key impacts on patients, caregivers, and families. Both conditions share key domains including seizures and problems related to neurological and motor function, behavior, communication, and sleep. These key areas provided the basis for evaluating existing COA measures and identifying domains of interest for assessment in a new measure. The conceptual models are provided in the supplementary materials (Figs. S1 and S2).

A literature review was conducted to determine if existing COA measures previously used in DS and/or LGS studies were fit for purpose to assess non-seizure symptoms. A shortlist of nine measures were identified for further evaluation: the Aberrant Behavior Checklist-Community (ABC-C); caregiver-completed version of the Vineland Adaptive Behavior Scale (VABS); Quality of Life in Childhood Epilepsy (QOLCE); Pediatric Quality of Life Inventory; Behavior Rating Inventory Executive Function; National Institutes of Health Toolbox Cognition Battery; Work Productivity and Activity Impairment questionnaire; Child Sleep Habits Questionnaire; and Anxiety, Depression, and Mood Scale.

Interviews with experts were held to discuss existing COA measures, which were evaluated in terms of content validity, sensitivity to change in status, and flexibility to assess improvement or worsening in individuals with profound intellectual disabilities.

Additionally, an item-level analysis was performed on data from caregiver-completed COA measures (the ABC-C, VABS, and QOLCE measures) used in the phase 2 ELEKTRA clinical trial (ClinicalTrials.gov: NCT03650452) in individuals with DS or LGS (data not published) [22]. The caregiver-completed exit survey was also analyzed. The exit survey included in ELEKTRA had previously been refined to produce an experience questionnaire used in the phase 2 ENDYMION 1 open-label extension trial (ClinicalTrials.gov: NCT03635073). Items from the experience questionnaire, and findings from the item-level analysis (of ABC-C, VABS, QOLCE, and the exit survey) were used to develop an initial draft of the CGI-I Non-seizure Symptoms measure.

### Delphi panel

To refine the draft CGI-I Non-seizure Symptoms measure, a three-round online Delphi panel was convened with clinicians and PAs. The goal was to reach a consensus on concepts relevant to DS and LGS and develop items that would adequately assess treatment impact in a heterogeneous population in each concept.

Clinician panel members were pediatric neurologists or pediatric epileptologists with relevant clinical expertise working with patients who had DS and/or LGS. Clinicians were identified from author lists of published literature in the disease areas of interest or from membership lists in disease-relevant consortia. PAs were representatives of a patient advocacy group for either DS or LGS and had experience working with both caregivers and patients. Five individuals previously interviewed as part of the conceptual model refinement were also invited to participate in the Delphi panel.

The Delphi panel was conducted using an online survey platform (Welphi v4.0) that allowed participants to complete the survey and view the responses/ratings of other participants after completion of the previous round. Three rounds of review were completed. In each round, participants rated each item and its description using the following scale: relevant and requires no revision; relevant but requires minor revision; relevant but requires major revision; or not relevant. Guided by the literature, the *a priori* established consensus threshold for progressing an item to the following round was ≥70% of panel members agreeing no revision or minor revision was required [23–25]. Participants could provide comments and suggest revisions as appropriate. After each round, quantitative rating data were analyzed using the same percentage agreement threshold, and qualitative data were reviewed.

To aid standardization and administration of the measure, an additional set of expert interviews and a two-round Delphi panel were also conducted with clinicians and PAs to develop and validate a standardized user manual for the CGI-I Non-seizure Symptoms measure.

### Cognitive debriefing interviews with caregivers

The final CGI-I Non-seizure Symptoms measure was debriefed with caregivers of individuals with DS or LGS as part of a qualitative interview study. Caregivers were recruited via advertisement by the advocacy organizations the Dravet Syndrome Foundation and Lennox-Gastaut Syndrome Foundation between January–March 2022. Prospective caregiver participants completed an online screening to determine their eligibility. To be included, individuals must have been a caregiver to a patient who had received a documented clinical diagnosis of DS or LGS and was aged 2–21 years (DS) or 2–35 years (LGS) at the time of informed consent. Caregivers were excluded if the individual they cared for was already enrolled in a clinical trial at the time of this study.

Following screening and consent procedures, hybrid interviews with caregivers that incorporated concept elicitation and cognitive debriefing were conducted by a trained interviewer via telephone or web-assisted audio; interviews were completed in one 90-min session or two 45-min sessions to accommodate caregiver preference. The brief concept elicitation was conducted to understand the patient’s seizure history, most problematic non-seizure symptoms, and impacts relating to these symptoms. To debrief the CGI-I Non-seizure Symptoms measure, the caregiver participants read the baseline descriptions and instructions for each item and used these descriptions to describe the patient’s current status. Their responses were analyzed to evaluate if each item was understood and relevant to their child. Participants ranked the importance of each item on a scale from 0 to 10 and discussed the rationale for their choice. Finally, caregivers were asked to reflect on what a hypothetical minimal improvement would look like in their child’s present condition using the item response scale, as well as if this amount of improvement would be considered meaningful to them, what impact this improvement would have, and to describe what a worsening of the patient would look like using the scale.

Interview transcripts were analyzed via a qualitative analysis plan; all qualitative data were coded and analyzed using the software package NVivo v13 (2020, R1) [26].

The study was conducted in compliance with good clinical practice guidelines, including International Conference on Harmonization guidelines [27]. All study documentation was approved by Western Independent Review Board (tracking number 20217011) before patient recruitment. All applicable laws and regulatory requirements were adhered to throughout the study.

## Results

### Preliminary analysis and draft measure development

Preliminary analysis revealed that although some items and/or domains in existing COA measures may be applicable to patients with DS or LGS, they are not fit for purpose for the measurement of non-seizure outcomes in these individuals. Key factors affecting the suitability of existing measures involve the heterogeneity of symptoms in DS and LGS, particularly varying levels of IDD, functional status, and verbal communication ability within these patient populations. An item-level analysis of COA measures included in the phase 2 ELEKTRA clinical trial (including the ABC-C, VABS, and QOLCE measures) identified floor effects for many items and a high level of missing data (data not shown). This analysis confirmed qualitative findings that some items in these measures were not fit for purpose in a population with IDD and thus would not be sensitive to change in a clinical trial. Additionally, some items were identified as being emotionally burdensome for caregivers of children with severe or profound IDD.

Findings from the ELEKTRA item-level analysis and exit survey analysis were utilized to refine items from the ENDYMION 1 experience questionnaire, which became the initial draft version of the CGI-I Non-seizure Symptoms and contained the following: verbal communication, nonverbal communication, receptive communication, alertness, social engagement, sleep disturbances, body movement, disruptive behaviors, hyperactive behaviors, anxious behaviors, attention span, negative mood, and positive mood (Fig. 1).

### Delphi panel

**Fig. 2:**
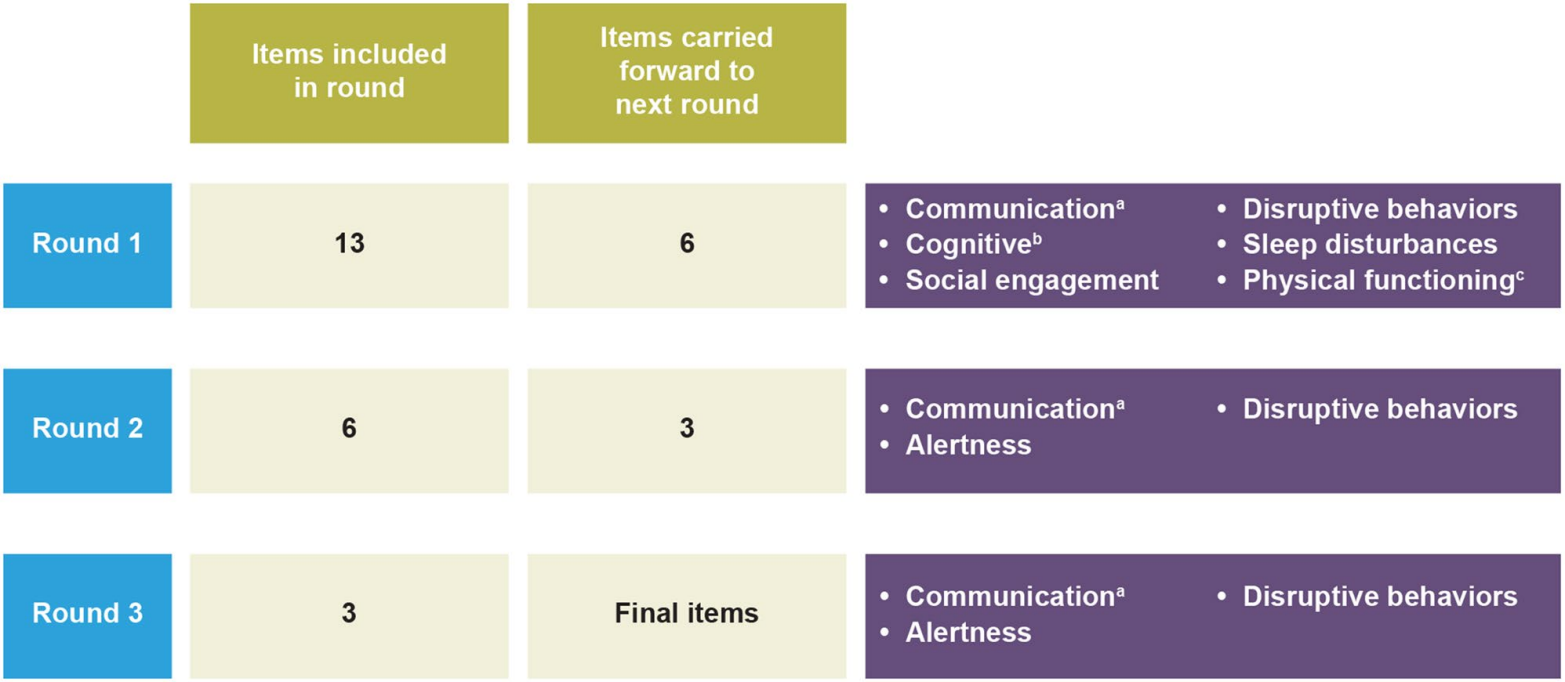
Delphi panel: refining the draft CGI-I Non-seizure Symptoms measure for individuals with DS and LGS. The *a priori* established threshold for consensus in the panel to keep an item was at least ≥70% of the panel agreeing that no revision or minor revision was required. ^a^Encompasses verbal communication and nonverbal communication in Rounds 1 and 2, with receptive communication added after Round 2. ^b^Encompasses receptive communication (removed after Round 2), attention span, and alertness (only alertness was carried forward after Round 2). ^c^Item added in addition to the five items agreed in Round 1. *CGI-I* Clinical Global Impression of Improvement, *DS* Dravet syndrome, *LGS* Lennox-Gastaut syndrome

The Delphi panel comprised 15 experts: seven epileptologists, two neurologists, and six PAs (Table 1). The initial 13-item draft measure was refined through three rounds to ensure appropriateness and relevance for use in DS or LGS clinical trials and to reduce item overlap. This resulted in a final measure comprising three items, intended to be scored independently as global measures of change from baseline. The outcomes of each round of the Delphi panel are summarized in Fig. 2 and detailed below.

**Table 1:** Demographic information for Delphi panel participants

	Clinician (n = 9)	Patient advocate (n = 6)^a^
Experience with DS^b^, n (%)	9 (100)	5 (83)
Experience with LGS^b^, n (%)	9 (100)	2 (33)
Years of experience, n (%)		
6–15 years	4 (44)	3 (50)
≥16 years	5 (56)	2 (33)
Clinical trial experience, n (%)	9 (100)	4 (67)

Percentages are subject to rounding

*DS* Dravet syndrome, *LGS* Lennox-Gastaut syndrome

^a^One patient advocate did not provide a response for these demographic questions. They were recruited via a patient advocacy group for DS, and therefore were assumed to have experience with DS

^b^Experience with both syndromes was not mutually exclusive for either group

#### Round 1

After Round 1, all 13 items met the established consensus threshold of ≥70% of panel members agreeing either no revision or minor revision was required; six items received a rating of “relevant and requires no revision” by >70% of panel members (verbal communication, 79%; nonverbal communication, 86%; alertness, 86%; social engagement, 71%; disruptive behaviors, 79%; positive mood, 79%).

When asked to rank items by importance, the following six items received the lowest support from panel members and were deemed least likely to be relevant: hyperactive behaviors (50%), body movement (50%), negative mood (43%), positive mood (43%), attention span (43%), and anxious behaviors (29%).

Revisions at this stage were aimed at reducing overlapping item content to lessen respondent burden and increase conceptual clarity. For example, verbal communication and nonverbal communication were combined into a single communication item. After individual item refinements based on panel suggestions, five items (communication, cognitive, social engagement, disruptive behaviors, and sleep disturbances) were progressed to Round 2. An item of physical functioning was added to the five items at this stage in response to comments from panel members that the measure should include an item assessing how activities of daily living are affected by DS or LGS. This item was intended to measure function rather than symptoms; therefore, it was presented to the panel separately from the other items in the CGI-I Non-seizure Symptoms measure.

#### Round 2

In Round 2, five of the six items reached the predetermined consensus threshold of ≥70% for the “requires no revision” or “requires minor revision” rating. Of these, three items had ≥70% consensus for “requires no revision”: social engagement (80%), disruptive behaviors (93%), and sleep disturbances (93%). The communication item was near ≥70% consensus for requiring no revision (67%), while the cognitive item had <60% of the panel responding with “no revision required” (53%). Although the sleep item was rated by all participants as important to the disease area, it was removed because sleep was to be addressed in another caregiver-completed measure included in planned phase 3 trials. As there are several suitable sleep scales available, removing the item was not considered an unmet need for this effort. The physical functioning item added at the end of Round 1 also met the threshold of ≥70% for the “no revision” or “minor revision” rating. However, this was the lowest-rated item, with only six panel members (40%) responding with “requires no revision”. Edits made to items resulting from Round 2 feedback included adding an explicit reference to “expressive” and “receptive” communication into the communication item, moving the receptive communication part of the cognitive item to the communication item, and moving alertness from the cognitive item into its own independent item.

#### Development of a user manual

Between Rounds 2 and 3 of the Delphi panel, interview feedback was gathered from five clinicians and one PA to guide the development of a CGI-I Non-seizure Symptoms measure user manual. All six interview participants had previously participated in the initial Delphi panel to validate the measure. Following the interviews, a two-round Delphi panel (Round 1: seven clinicians, five PAs; Round 2: nine clinicians) was conducted to refine and gain consensus on the user manual content. This feedback was also used to make further revisions to the measure item content before Round 3. Clinicians supported the combination of receptive and expressive communication items into a single item because this allowed for the communication item to be catered to each patient’s baseline (e.g., those more severely impaired may still make receptive communication improvements even if they do not demonstrate improvements in expressive communication). After these amendments, three items were moved forward into Round 3: communication, alertness, and disruptive behaviors.

#### Round 3

In Round 3, ≥70% of panel members agreed each of the three reviewed items required “no revision” (communication, 86%; alertness, 100%; disruptive behaviors, 100%), surpassing the predetermined consensus threshold of ≥70% for the “no revision” or “minor revision” rating required, confirming these three items as components of the final CGI-I Non-seizure Symptoms measure.

### Caregiver interviews

In total, 21 caregivers took part in interviews to debrief the CGI-I Non-seizure Symptoms measure (10 caregivers for patients with DS and 11 caregivers for patients with LGS). All caregivers were female parents with a mean age of 41 years (range 33–57 years). Most individuals with DS or LGS were male (61.9%) with a mean age of 10 years (DS: 7.3 years, LGS: 12.7 years). These individuals had a range of communication and ambulatory abilities (Table 2).

**Table 2:** Demographic information for caregiver interview participants and the patients they care for

		DS	LGS
		Recruited (n = 10)	Recruited (n = 11)
Caregiver education, n (%)	High school diploma or below	0 (N/A)	2 (18)
Age of patient, n (%)	2–5 years	3 (30)	2 (18)
	6–10 years	6 (60)	2 (18)
	11–15 years	1 (10)	3 (27)
	16–21 years	0 (N/A)	3 (27)
	22–35 years (LGS only)	N/A	1 (9)
Ability of patient to walk, n (%)	Walks independently	8 (80)	5 (45)
	Walks with assistance or a device	2 (20)	3 (27)
	Not able to walk	0 (N/A)	3 (27)
Ability of patient to talk, n (%)	Verbal	8 (80)	4 (36)
	Nonverbal	2 (20)	7 (64)

Percentages are subject to rounding

*DS* Dravet syndrome, *LGS* Lennox-Gastaut syndrome, *N/A* not applicable

#### Concept elicitation

The interviews provided insights into each individual’s seizure history, the most problematic symptoms of each condition, and the associated disease impacts experienced by affected individuals, caregivers, and other family members. The non-seizure symptoms discussed by most caregivers were neurological and motor impairment (n = 21/21 [100%]), communication issues (n = 15/21 [71%]), and behavioral issues (n = 13/21 [62%]). Interview responses provided spontaneously during the concept elicitation aligned with the key features and impacts of DS and LGS we identified in the previously developed conceptual models for both DS and LGS (Figs. S1 and S2), further supporting the validity of the measure items.

#### Item understanding, relevance, and importance

**Fig. 3:**
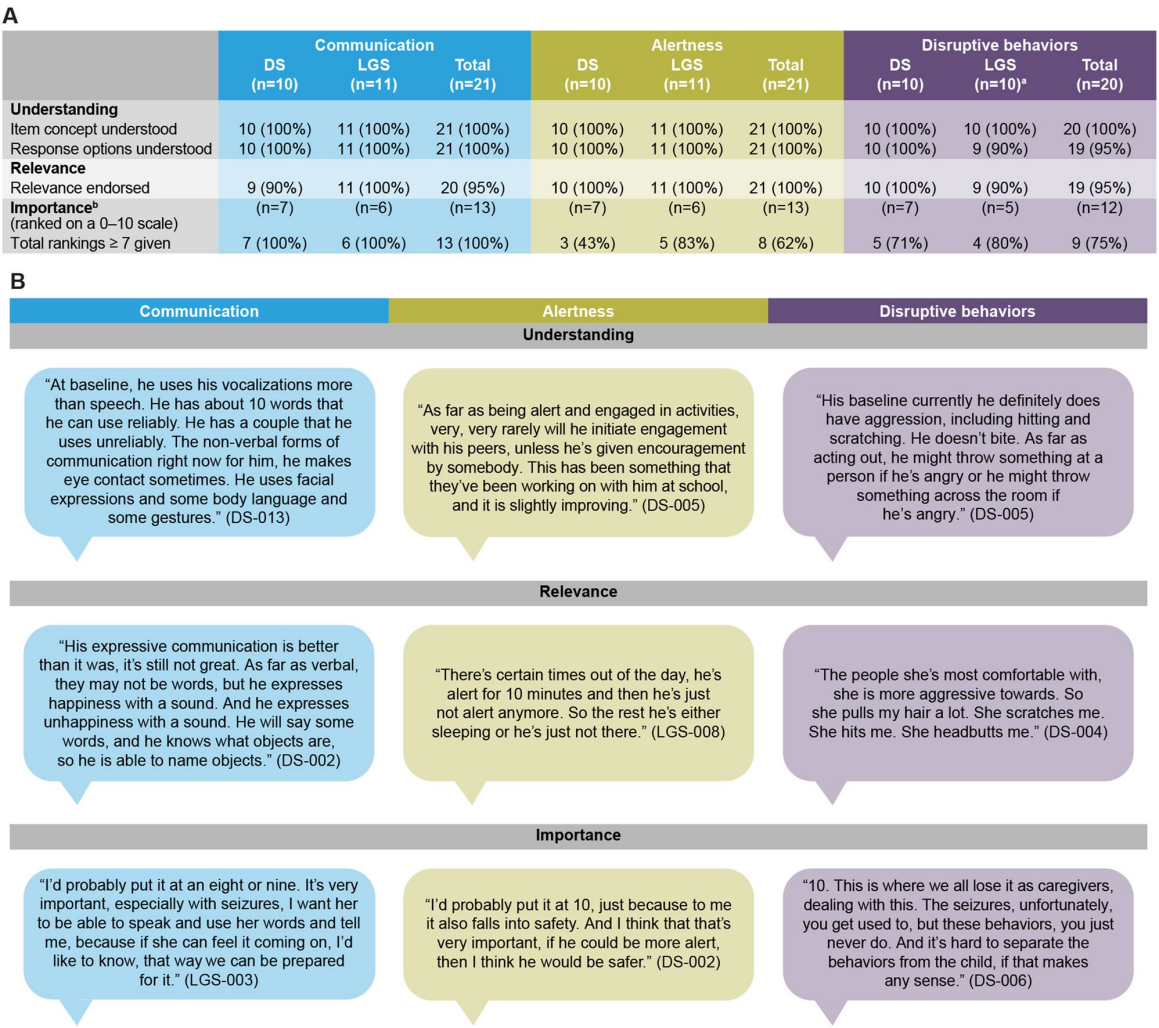
Caregiver reports for the three CGI-I Non-seizure Symptoms measure items (**A**) with caregiver quotes (**B**). ^a^The disruptive behaviors item was not discussed with one caregiver because it was deemed not relevant given the severe nature of their child’s condition. ^b^Some caregivers were asked to discuss item importance without explicitly providing an importance ranking, while others were not asked about item importance owing to time constraints. Quotes have been edited for clarity. *CGI-I* Clinical Global Impression of Improvement, *DS* Dravet syndrome, *LGS* Lennox-Gastaut syndrome

All caregivers who were asked demonstrated they could understand the item concept from the baseline item descriptions, use these descriptions to describe their child’s status (communication, n = 21/21 [100%]; alertness, n = 21/21 [100%]; disruptive behaviors, n = 20/20 [100%]), and use the item response scale to describe levels of change or improvement for their child (communication, n = 21/21 [100%]; alertness, n = 21/21 [100%]; disruptive behaviors, n = 19/20 [95%]) (Fig. 3). One caregiver of an individual with LGS was not debriefed about the disruptive behaviors item owing to the severity of their child’s condition.

Almost all caregivers reported that the concepts captured by each item were relevant for their child with DS or LGS (alertness, n = 21/21 [100%]; communication, n = 20/21 [95%]; disruptive behaviors, n = 19/20 [95%]). Caregiver responses demonstrated that the item descriptions were sufficient to allow caregivers to describe their child’s current status, which further indicated both caregiver understanding and relevance of the items. For alertness, interpretation differed slightly among caregivers but aligned with the severity of their child’s condition. Of the 13 caregivers who were asked in the interviews to directly rate the importance of the items on a scale from 0 to 10, the majority ranked each item as ≥7 (communication, n = 13/13 [100%]; disruptive behaviors, n = 9/12 [75%]; alertness, n = 8/13 [62%]). Importantly, nearly a quarter or more of those who responded rated the items as 10/10, indicating these were critical areas of importance (communication, n = 5/13 [38%]; disruptive behaviors, n = 4/12 [33%]; alertness, n = 3/13 [23%]). Caregiver quotes supporting the understanding, relevance, and importance of each item are provided in Table S1.

#### Amount of improvement in each item required for meaningful change

**Fig. 4:**
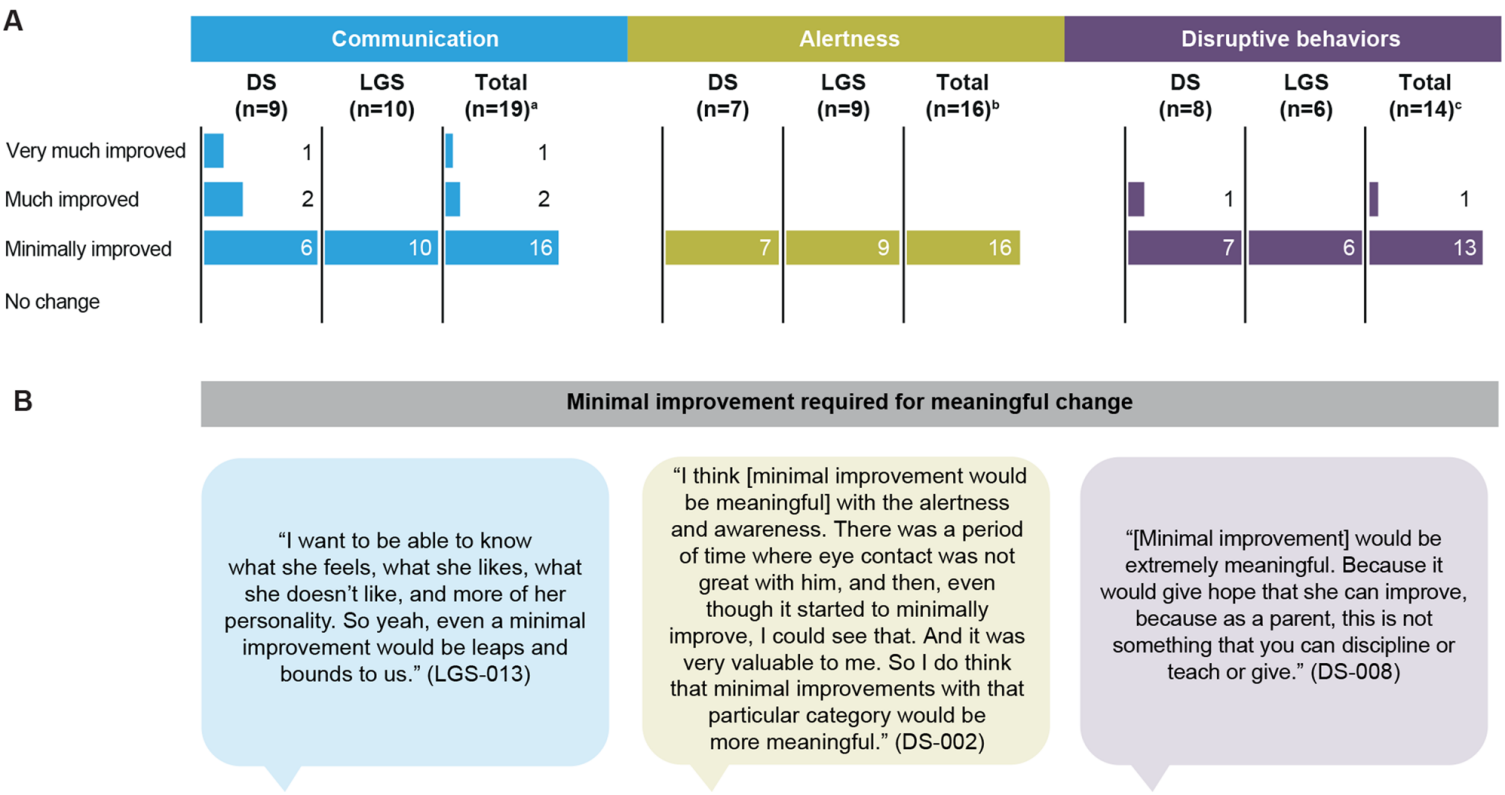
Caregiver-indicated minimum improvement needed for meaningful change to them and their child in each item (**A**) with caregiver quotes (**B**). ^a^n = 2 were not included because an answer could not be determined from the discussion. ^b^n = 4 did not provide data on whether minimal improvement would be meaningful; n = 1 indicated that it was probably meaningful but was not explicit. ^c^n = 1 did not discuss the item because it was not relevant owing to the severity of their child’s condition; n = 4 were not asked if a change would be meaningful; n = 1 did not experience disruptive behaviors; n = 1 did not experience disruptive behaviors and discussed that having “no change” would be good. Quotes have been edited for clarity. *DS* Dravet syndrome, *LGS* Lennox-Gastaut syndrome

For each item, most caregivers reported a minimal improvement would represent a meaningful outcome to them and their child (alertness, n = 16/16 [100%]; disruptive behaviors, n = 13/14 [93%]; communication, n = 16/19 [84%]) (Fig. 4). Caregiver quotes highlighting specific examples of minimal improvement are provided in Fig. 4 and Table S2.

## Discussion

Our review found that the available epilepsy-specific pediatric COA measures are not fit for purpose for individuals with varying levels of IDD and heterogeneous disease presentation, such as those with DS or LGS. As such, an unmet need exists for disease-specific measures that can be used to assess non-seizure symptoms in clinical trials. In this study, we used a global impression approach to develop a novel ClinRO measure, the CGI-I Non-seizure Symptoms measure, that addresses the limitations of existing COA measures for assessing within-patient change in individuals with DS or LGS. Preliminary conceptual model development identified shared symptom complexes between DS and LGS (except an autonomic domain specific to DS), and so a single measure was considered to be appropriate for both conditions. The three key domains captured by the final measure (communication, alertness, and disruptive behaviors) were found to be relevant and important by expert clinicians, PAs, and caregivers of individuals with DS or LGS.

To adapt the CGI measure, consultation with expert clinicians was required to ensure that measure content is usable, relevant, and expected to be sufficiently sensitive to detect change in a clinical setting. Moreover, because the CGI-I Non-seizure Symptoms measure was developed to be completed by clinicians with input from caregivers, assessing caregiver feedback on the relevance and understandability of the items was also important. In this study, we sought feedback from both caregivers and clinicians. In the first stage, we refined the CGI-I Non-seizure Symptoms measure through a Delphi panel with expert clinicians and PAs. Through this process, an initial draft set of 13 items was refined to three final items: communication, alertness, and disruptive behaviors. Based on preliminary work, these three items appear to be among the most burdensome non-seizure symptoms in individuals with DS or LGS, and are considered by both clinicians and caregivers to be important to assess.

Measuring cognitive improvement in a low-functioning population presents considerable challenges [11], and alertness is an observable proxy that may be foundational for improvement in all other areas. Communication and disruptive behaviors were also considered to be linked to cognitive function [12]; thus, these three areas of observable behavior associated with the conditions could be assessed as alternative indicators of change in cognitive functioning [28].

Collaboration between clinicians and caregivers is a key component to supporting effective use of the CGI-I Non-seizure Symptoms measure. In the cognitive debriefing with caregivers of patients with DS or LGS, responses demonstrated that the three CGI-I Non-seizure Symptoms measure items were easy to interpret and represented relevant non-seizure outcomes important for patients with a broad range of symptoms and disease severity. These responses were consistent with the conceptual models for the key features and impacts of DS and LGS. These findings provide evidence to support the use of the CGI-I Non-seizure Symptoms measure in clinical studies, in which trial clinicians, through discussion with and with the assistance of the primary caregiver, can establish a baseline description of the study participants’ condition before treatment initiation. This baseline description can then be used to inform assessment of change at subsequent study visits. The benefit of this inherent collaboration with caregivers will result in clinicians obtaining a more comprehensive impression of changes over time, rather than relying on observations made during that particular assessment [29, 30].

A variety of approaches for implementing global impression scales have been observed in contemporary clinical trials of patients with DS or LGS. A single overall CGI-I measure has previously been used in phase 3 studies of fenfluramine (in patients with LGS) [31] and cannabidiol (in patients with drug-resistant epilepsies, predominantly DS and LGS) [32]. Both studies included clinician and caregiver CGI-I ratings as secondary outcomes. Although clinician and caregiver ratings were compared to assess improvement from baseline resulting from treatment, whether CGI-I assessments were completed collaboratively between clinicians and caregivers was not reported. In both studies, caregiver-rated CGI-I scores were generally higher than physicians’ scores [31, 32]. In the cannabidiol study, caregiver and physician scores were significantly correlated (*p* < 0.001), with both sets of scores increasing over time [32]. Future clinical trials should consider collecting global impression responses from a variety of reporters to provide a diverse understanding of the patient experience.

Establishing an understanding of the smallest change in a COA that is perceived as important can assist with interpreting the value of a treatment or establishing a meaningful change threshold for COA measures [33]. In the present study, most caregivers reported even a minimal improvement in each of the three domains would have a meaningful impact for them and their child. These findings further support the importance of the measure items as non-seizure outcomes.

A strength of this study was the systematic approach to gathering feedback from both clinical experts and caregivers of individuals with DS or LGS. This has resulted in a final measure that is relevant and appropriate for use in these conditions and can be completed by clinicians with assistance from caregivers. Additionally, the sample included caregivers of those with varying cognitive, communication, and ambulatory abilities and thus reflects the heterogeneity observed within DS and LGS populations.

A limitation of the study was that the measure was developed in US English and feedback on it was received primarily from clinicians and PAs who were native English speakers based in the USA, with one clinician in the UK. Moreover, the cognitive debriefing was undertaken with English-speaking US-based caregivers only, which may limit the representativeness of the findings.

An additional limitation of the study was that not all feedback on the items from the Delphi panel or caregiver interviewers could be addressed. Although changes were made to measure items based on Delphi panel results, contradictory feedback was received at times. As such, it was not possible to tailor the measure based on every response received. Several queries and suggested changes for the measure items also arose during the caregiver interviews. These tended to be proposed by a single caregiver, were not consistent between caregivers, or were already covered by the further guidance provided in the user manual. This further emphasized the need for trial clinicians to receive systematic training in the use of the measure, and for them to refer to the user manual to guide their notes and assessment when completing the measure.

Finally, this study was undertaken during the COVID-19 pandemic, which may have influenced discussions, particularly with regard to disruptive behaviors and sleep disturbances, which were found to have been affected by lockdowns relating to COVID-19 in individuals with DS [34] or LGS [35], and in children with epilepsy [36].

## Conclusions

The final CGI-I Non-seizure Symptoms measure is designed to assess changes in three key domains (communication, alertness, and disruptive behaviors) defined as relevant and important by expert clinicians, PAs, and caregivers of individuals with DS or LGS. This measure is the first COA measure specifically tailored for use with individuals with DS or LGS, and the process of refinement and validation by clinicians and caregivers described here supports the use of the measure to be reliably used in a clinical trial setting. It is our hope that this measure will enable researchers to evaluate within-patient change in domains beyond seizure frequency, and ultimately benefit individuals with DS or LGS and their caregivers. Further work to evaluate the measure is planned following completion of three ongoing phase 3 studies (ClinicalTrials.gov: NCT04938427, NCT04940624, NCT05163314).

## Electronic supplementary material

Below is the link to the electronic supplementary material.

Supplementary Material 1

## Data Availability

The datasets, including the redacted study protocol, redacted statistical analysis plan, and summary data supporting the results, will be made available after the publication of study results within three months from initial request to researchers who provide a methodologically sound proposal. The data will be provided after its de-identification, in compliance with applicable privacy laws, data protection and requirements for consent and anonymization.

## Abbreviations

ABC-C: Aberrant Behavior Checklist-Community
CGI: Clinical Global Impression
CGI-I: Clinical Global Impression for Improvement
ClinRO: Clinician-reported outcome
COA: Clinical outcomes assessment
DS: Dravet syndrome
IDD: Intellectual and developmental disability
LGS: Lennox-Gastaut syndrome
PA: Patient advocate
QOLCE: Quality of Life in Childhood Epilepsy
VABS: Vineland Adaptive Behavior Scale
