# Supplementary material for: Development and refinement of the Clinical Global Impression of Improvement for Non-seizure Symptoms measure in Dravet syndrome and Lennox-Gastaut syndrome
Source: J Patient Rep Outcomes. 2025 Feb 21;9:24. doi: 10.1186/s41687-024-00829-2 (PMC11845656; doi:10.1186/s41687-024-00829-2)
Supplement: Supplementary file 1 — Supplementary Material 1 [file 41687_2024_829_MOESM1_ESM.docx]

# Supplementary material


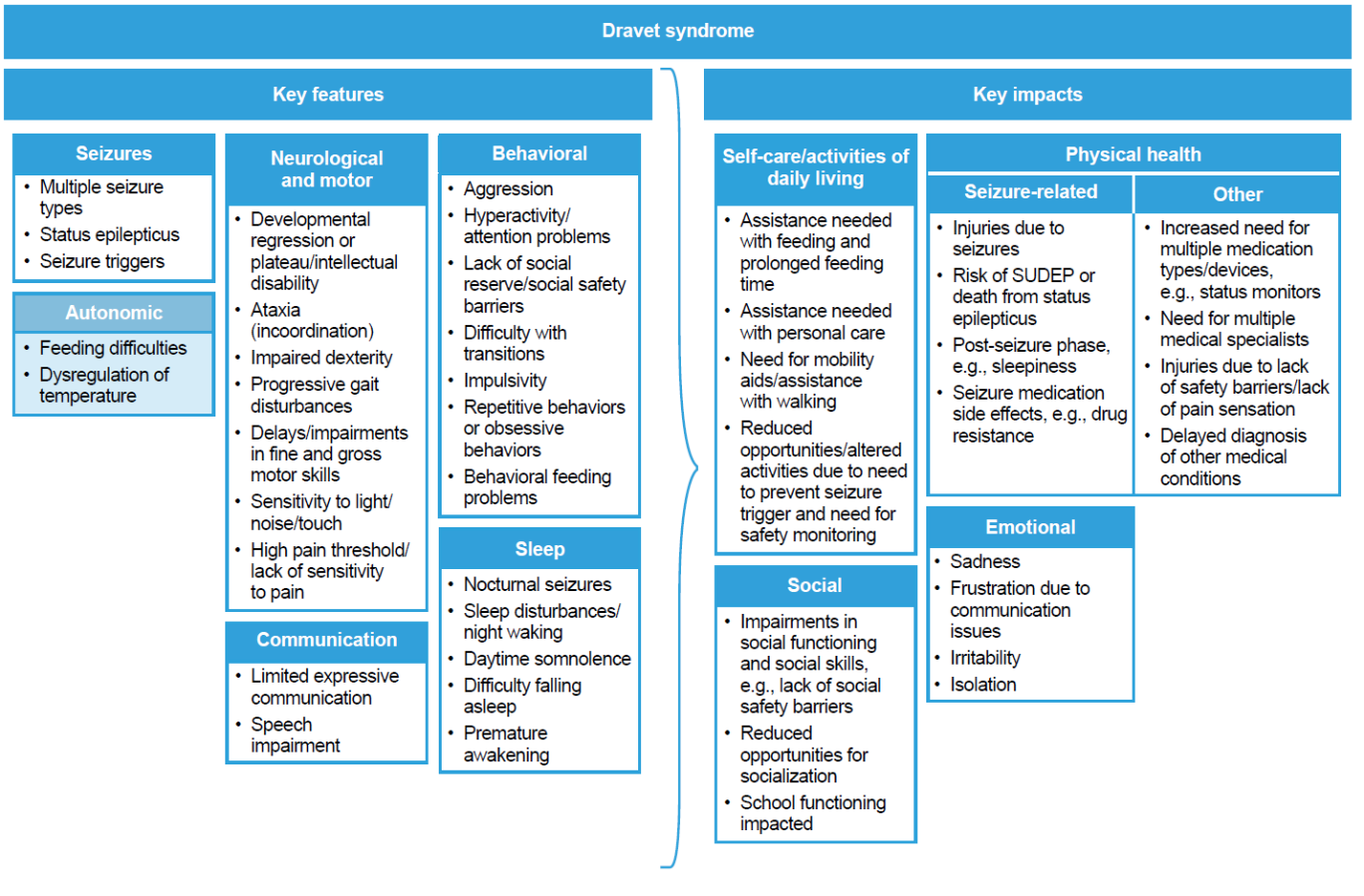


*SUDEP*, sudden unexpected death in epilepsy.

Fig. S1 Conceptual model of key features and impacts of Dravet syndrome


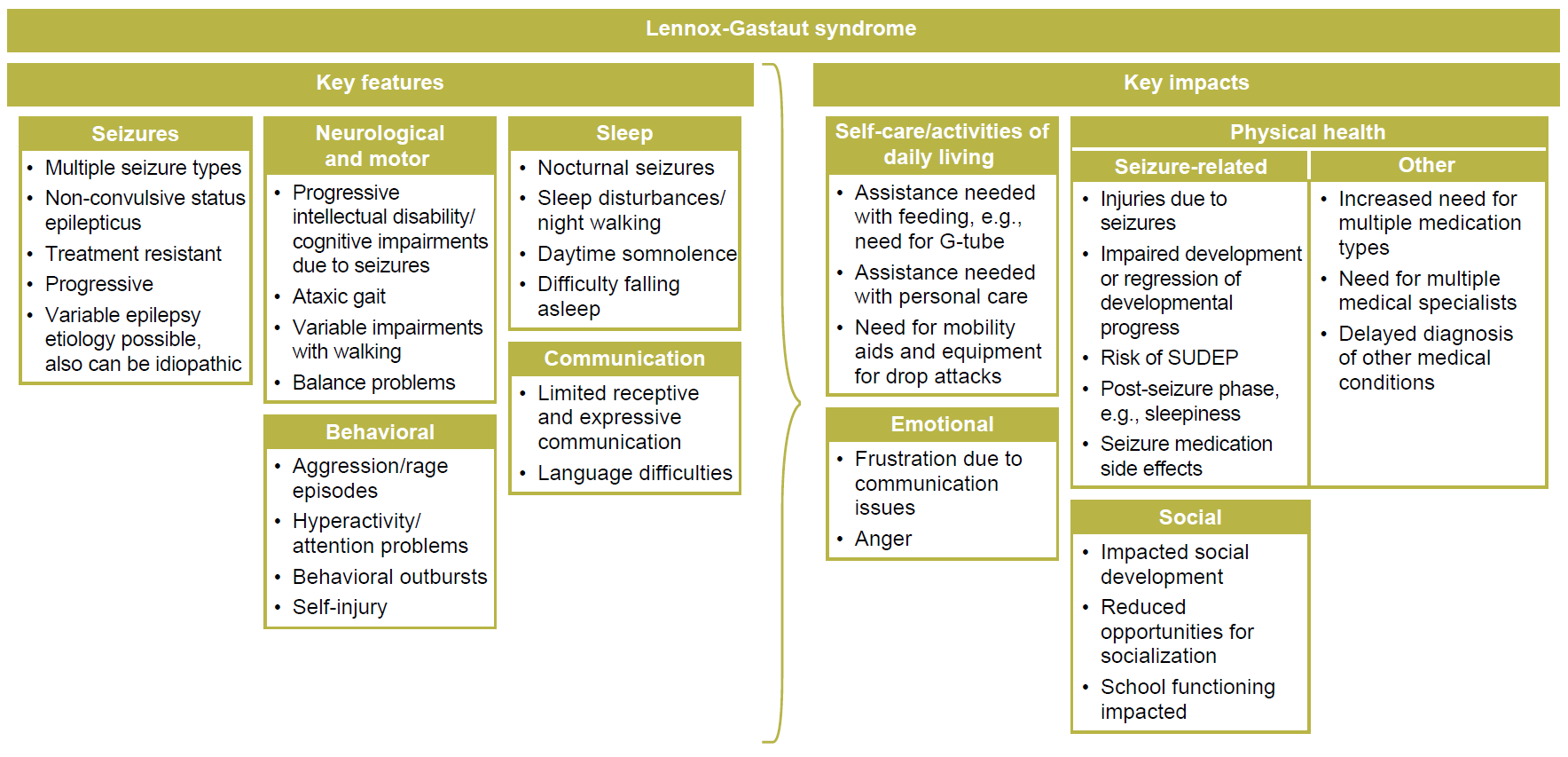


*SUDEP*, sudden unexpected death in epilepsy.

Fig. S2 Conceptual model of key features and impacts of Lennox-Gastaut syndrome

**Table S1** Caregiver quotes supporting item understanding, relevance, and importance

| **CGI item** | **Concept** | | |
| --- | --- | --- | --- |
|  | **Understanding** | **Relevance** | **Importance** |
| Communication | “Okay. So, um, at baseline, he uses his vocalizations more than speech. Um, he has about 10 words that he can use reliably. He has a couple that he uses unreliably. Um, the nonverbal forms of communication right now for him, he makes eye contact sometimes. Um, he uses facial expressions and some body language and some gestures.” (DS-013)  “Um, I would say as far as expressive communication, um, he definitely, um, would first use, um, gestures, body language, um, that kind of stuff to tell us what he wants either, you know, by pointing what he wants or grabbing our hand and bringing us to what he wants.” (DS-005)  “He's pretty good at eye contact. Um, I mean, sometimes, he does it, but then sometimes you can definitely get him to look you in the eyes … Um, he definitely has an animated face. So he has, his facial expressions. Um, that's mostly how I can tell, how we can tell, you know, how he feels or if something's about to happen.” (LGS‑007)  “So, but her receptive communication skills would be pretty, pretty low. I wouldn't, um, I wouldn't qualify it as, like, being able to follow instructions or respond to questions at all.” (LGS-013)  “But, um, you know, he can follow instructions if it's one or two steps that he's familiar with. Um, you know, if we came up with some totally random instruction that he had never done before, he probably would not be able to do it. Um, but if we ask him questions, he can, um, nod his head or say yes or shake his head and say no.” (DS-005) | “Um, so his expressive communication is, it’s … it’s better than it was, it’s still not um, great. But uh, as far as, you know, verbal, they may not be words, but he expresses happiness with a sound. And he expresses unhappiness with a sound. Um, he will say some words, and he knows what objects are, so he, he is able to name objects.” (DS-002)  “Um, it's very jumbled and … Poorly pronunciated, um, but that's why we have the talker so she can use that in assistance too.” (LGS-003)  “Um, I'd say her ability to do the receptive communication is very low. Um, um, she responds to voices. Um, and, like, in her physical therapies and stuff, it, it's, we have to show her what we want her to do, so it's not really just tell her, it's, we have to kind of show her and, and coerce her physically to do it. So, but her receptive communication skills would be pretty, pretty low. I wouldn't, um, I wouldn't qualify it as, like, being able to follow instructions or respond to questions at all.” (LGS-013)  “… she expresses her needs by like, she doesn't vocalize her, vocalize, but it's more in the terms of like crying out, she doesn't use words. Um, she, she will reach for me, so I guess that's a gesture. She really doesn't make eye contact, but her, I can tell when she needs me, usually 'cause she's crying and reaching for me.” (DS-004) | “Oh, I’d probably put it at, like, an eight or nine. I mean, it’s very important, um, especially with seizures, um, I want her to be able to speak and use her words and tell me, because if she can feel it coming on, I’d like to know, that way, we can be prepared for it.” (LGS-003)  “10, 10, 10, 10 … Because I hate the times where they’re not able to tell you where it hurts or what's happening to them … I love that he's able to come to me and say, ‘the seizure's coming. I feel one coming. I'm hungry. I need to go to the bathroom. I want to sleep with you. I love you.’” (DS-009)  “I would put it at 10 … Um, because they have such specific needs, I feel it's important that they have the communication skills to be able to express what's bothering them, um, and, and tell you their needs.” (DS-006)  “Um, I would put it probably about like a nine-ish … I feel like if she was able to better communicate that, you know, she would just overall be happy. I hate that she's so unhappy, and I can't imagine what it's like to not be able to express yourself.” (DS-004)  “Oh, um ... 10 … In any way. You know, I don't ... I'm always saying like communication for me is important. Uh, you don't have to talk, just, you know, just trying to ... A way to communicate, if it's sign language or showing me, but it is important.” (LGS-009) |
| Alertness | “Um, and as far as, you know, being alert and engaged in activities, um, very, very rarely will he initiate engagement, um, with his peers, um, unless he’s given encouragement by somebody. This has been something that they’ve been working on with him at school, um, and, you know, it is slightly improving.” (DS-005)  “Okay. He is 100% aware and-and attentive of surroundings. Um, an example of that would be I'm frantically looking for my car keys or my phone or whatever and he is like, ‘Hey mom, it's on your dresser in your bedroom.’” (DS‑003)  “Um, he is alert and attentive to his surroundings in his comfortable environment, like when he knows where he's at, like we're at home … Um, and you know, he knows like if I move the furniture around, you know, you can see that oh, he typically goes this way and he stops and he looks, and he's like, ‘Wait, now I have to go a different way, or I have to do a different thing.’ So I do feel like he's alert and attentive to his surroundings.” (DS-002)  “He's, he's very alert and engaged with adults. Um, like, if they're sitting with him and he wants them to look at a book, like, he wants your undivided attention. So when it's something that he, it's important for him, he's, you know, very alert and engaged.” (LGS‑007)  “Um, and that she also gets hyper focused. Like she, she has an iPad that she likes to watch videos on. And she gets so like focused on that, that you know, the, the house could be on fire and she wouldn't be aware, um, 'cause she's just so focused on that, that favorite thing right there. So, the awareness is not, not quite there.” (LGS-010) | “There's maybe certain times out of day, he's just alert for like 10 minutes and then he's just, he's just not alert anymore, so … So the rest he's either sleeping or he's just not, not there.” (LGS-008)  “… if he was walking out to the car by himself, would he remember to look both ways or like look around to make sure nobody was following him? Probably not…and he's not going to necessarily look and say, ‘Okay, well, if I fell here and had a seizure, would I hit my head on this coffee table?’ He's not ... He wouldn't think of that, right?” (LGS-005)  “... she's generally alert through the day, but has periods, especially after a seizure where she's either unconscious or just like zoned out. Um, and that she also gets hyper focused. Like she, she has an iPad that she likes to watch videos on. And she gets so like focused on that, that you know, the, the house could be on fire and she wouldn't be aware, um, 'cause she's just so focused on that- that favorite thing right there.” (LGS-010)  “… we've had periods of time where he can't even attend to a task for longer than 10 seconds where his eyes are moving, his head is moving, he's focused on something else.” (DS-013)  “And then as far as attentiveness, she has the, her attention span is almost nonexistent. Um, so whenever we work with her, we usually have to, to put her in a high chair and eliminate like all other stimuli that we can, for instance the TV.” (DS-004) | “Um, I’d probably put it at 10, just because it, to me, it also falls into safety. And I think that that’s very important, if he could be more alert, then he would be more ... I think he would be safer.” (DS-002)  “I would say 10, because again, we, we could be talking about, um, we could be talking about, uh, more seizure activity. You know, if he's not as alert, okay, well, is it just because he didn't sleep well or is he having more seizures?” (LGS‑004)  “Um, I'll give it a nine … Because I, I, I feel bad for her when she's not able to really enjoy herself. So if she's able to be more alert, she'll be able to enjoy life a little better … I don't know, like the little things, you know, she'll be more into her toys or just more, um, if you're playing with her, she'll be more engaged.” (LGS-012)  “Um, probably a five … Um, as I said before, you know, um, obviously it's important, definitely like I want her to be able to progress, um, as far as, you know, her alertness or attentiveness for sure. However, I feel like there are more important things that, that, that would be more beneficial to her.” (DS‑004) |
| Disruptive behaviors | “Um, his baseline currently he definitely does have aggression, um, including hitting and scratching. Um, he doesn’t bite. Um, as far as acting out, he might throw something at, um, a person if he’s angry or he might throw something across the room if he’s angry.” (DS-005)  “Um, she has all the ones listed below except self-injury. She's never really done anything like that. Um, she's had aggressive tendencies, um, hitting, acting out, throwing things has been a big thing for her, um, screaming, yelling, tantrums, crying, fussing, whining, um, she gets fixated on things. She's gets obsessed with things, phrases, um, ways things have to be…” (DS-008)  “Um, but he, he is oppositional and defiant. He will say, no, like I said earlier, he'll just kind of turn his, it's almost like he's rolling his eyes. He just kind of turns his head and like, doesn't look at you. If he ... If you ask him to do something, he doesn't wanna do it. Um, his big one at the moment within like the past couple weeks has just been screaming, ‘No,’ when he doesn't wanna do something and really like yelling it.” (DS-006)  “And the biting, yeah. It's, it's just ... so we have to carry this like little bracelets, uh, so she doesn't go through her skin. Um, we carry chew tubes. Uh, we carry a whole bunch of things to try to soothe her from all these, um, behaviors that she's developed. And, um, it's just time consuming on top, on top of everything else that we have to deal with.” (LGS‑012) | “She, uh, the people she’s most comfortable with, she is, uh, more aggressive towards. So she, she pulls my hair a lot. She scratches me. She hits me. She headbutts me.” (DS-004)  “Um, if she's frustrated for some reason, she can also pinch. Um, so pinching, especially at the neck is really not fun. Um, so the headbutting and the pinching, uh, when she's frustrated, I would say that, that's probably the problematic thing.” (LGS-010)  “Oh, this is huge for us. So he's aggressive. He hits his peers, and sisters, and us. He kicks everyone. He has this stereotype that he raises his hands and he looks like a crazy person. He doesn't self-injure. He has temper tantrums. He has oh, fixations and objections a gazillion … Oh my God, we have over a thousand items of cups in America, in our house. The only sport that we watch is baseball. And the only thing that he plays is baseball.” (DS-009)  “Um, we have aggression with scratching and acting out. We have self-injurious behaviors daily. We have, um, screaming and crying if he doesn't like or want to do something, um, or if we're inter, trying to introduce something new to him, he does not tolerate that. Um, he has fixations on his iPad with videos and songs and portions of songs that he repeats over and over and over again and wants us to pay attention to.” (DS-013)  “…if he gets frustrated or mad about something or someone he does this thing where he just balls up his fists and sometimes he'll kind of like bang his fist either on his legs or on a table or something that he's, he's nearby.” (LGS-001)  “… like if we're in the grocery store and I tell her, ‘No, you can't get a toy,’ she sits there and she'll scream. And then, of course, it's kind of embarrassing, 'cause everybody's looking at you and you're like, ‘Come on, let's go.’ And she goes, ‘No!’ And, like, sits there, it's, it's, um, very frustrating as an adult, where you, you know, you're supposed to have your kids listen to you.” (LGS-003) | “10 […] this is where we all, we all lose it as caregivers, as dealing with this. I mean the seizures, unfortunately you get used to, but these behaviors, you just never. And it’s hard to, it’s hard separate the behaviors from the, from the child, if that makes any sense …” (DS-006)  “Um, I would say that's probably a seven or eight just because the more he does that, the less people want to, want to be around him and that's just not a fun life for anybody (laughs) … he will never live on his own, he will always be dependent on caregivers, um, and, you know, when you, if you're aggressive, especially physically aggressive, that limits the number of people who are willing to, you know, work with you and help take care of you and that kinda stuff.” (DS-005)  “Um, I would put it in about, um, like I said, like a seven. Um, because obviously, like I said before, there are some things that I feel like are more important and some things that I think ... like for instance communication, um, I would put communication above this. Because … improved communication would help with this.” (DS-004)  “Um, I don't know, I'd probably say a six or a seven. It's, it's up there, but it's not priority. Um, she doesn't need this, she doesn't need to control her temper tantrums and her crying in order t, to survive or to get through day to day, I feel. Um, I feel communicating and stuff like that will. It's more important …” (LGS-003) |

**Table S2** Caregiver quotes – smallest improvement required for meaningful change

| **CGI item** | **Meaningful change** | | |
| --- | --- | --- | --- |
|  | **Minimally improved** | **Much improved** | **Very much improved** |
| Communication | “Um, so yeah, it would be, even a minimal improvement would be leaps and bounds, um, to us.” (LGS-013)  “Um, a minimal improvement, I would say, would be learning a handful of new requests, like learning to s, vocalize, verbally say, um, five to 10 new requests. Um, maybe, uh, being more willing to have back and forth conversation, even if it's shorter … So even just an attempt at that would be a … minimal improvement that, you know, would be exciting for us.” (DS-005)  “I do [think minimal improvement would be meaningful], 'cause I think any change would be- would be a positive change. I think no change or anything below that would be ... Yeah, I would- I would be happy just to see slight improvement, so ...” (DS-007) | “Um, just in a way that, whether it was verbal or nonverbal, but just being able to communicate his needs better would be much improved … Uh, yes. Definitely [would be meaningful] … And so kinda having those needs met through communication I think would be um, just uh, a great gain.” (DS-002)  “… meaningful change for me would be that he, um, responds with meaningful language … But if I ask him if he's, um, hungry that I can rely on what his answer is … Yeah, I would say that [this was counted as much improved]” (DS-013) | “For [child’s name], it would have to be, uh, very much improved … because whenever you think about putting your child in a clinical trial, you're going to have a lot of considerations … In order for us to take up on this, it would have to be like a very much improved thing.” (DS-003) |
| Alertness | “Um, to take cues from how other people around her are approaching a situation, or learn, um, why certain, learn that certain things are dangerous … [That would be] Extremely meaningful, because if you can stop and think then that would apply, that would, um, blend with and affect every area of your life.” (DS-008)  “If it means that he would hurt himself less or be a little more safe, then maybe we would take that minimal improvement with safety as far as alertness and awareness.” (DS-002)  “Um, minimally improved would be if he's taking in the environment around him, so maybe pointing out things that he wants to see or being engaged in, kind of drawing our attention to it … it would be a meaningful improvement.” (DS-013) | - | - |
| Disruptive behaviors | “I would say instead of the five, or the five to 10 minutes, uh, maybe we move down to two minutes of a meltdown, or maybe we still cry and we pout, but we keep walking through the store. Um, like, that would probably be minimal improvement for me … Yeah [it would be meaningful], 'cause I could get my grocery shopping done a lot faster. (laughs)” (LGS-003)  “Not hitting, not grabbing, be able to go to the park, not having to have an adult follow him … Oh yeah, very. [meaningful].” (DS-009)  “I feel like any, any change in the right direction with, with, with these behaviors would be, uh, an improvement … Yes [any positive improvement would be a meaningful change]. This is where we struggle most with [patient’s name] is, is these disruptive behaviors.” (DS-006) | I feel like much improved would show us that he's able to control the urge enough that he, like, knows what he's doing … it gives you more hope and it gives you more like, ‘Okay, maybe we can redirect this and show you like, that you can still express this thing, but do it this way.’” (DS-002) | - |
